# Open water dreissenid mussel control projects: lessons learned from a retrospective analysis

**DOI:** 10.1038/s41598-023-36522-5

**Published:** 2023-06-27

**Authors:** Angelique D. Dahlberg, Diane L. Waller, David Hammond, Keegan Lund, Nicholas B. D. Phelps

**Affiliations:** 1 Minnesota Aquatic Invasive Species Research Center, University of Minnesota, 135 Skok Hall, 2003 Upper Buford Circle, St. Paul, MN 55108 USA; 2 Department of Fisheries, Wildlife and Conservation Biology, College of Food, Agriculture and Natural Resource Sciences, University of Minnesota, 135 Skok Hall, 2003 Upper Buford Circle, St. Paul, MN 55108 USA; 3 U.S. Geological Survey, Upper Midwest Environmental Sciences Center, 2630 Fanta Reed Road, La Crosse, WI 54603 USA; 4 Earth Science Laboratories, Inc, 903 N 47th Street, Suite 105, Rogers, AR 72712 USA; 5 Minnesota Department of Natural Resources, 500 Lafayette Road, St. Paul, MN 55155 USA

**Keywords:** Invasive species, Invasive species

## Abstract

Dreissenid mussels are one of the most problematic aquatic invasive species (AIS) in North America, causing substantial ecological and economic effects. To date, dreissenid mussel control efforts in open water have included physical, biological, and chemical methods. The feasibility of successful dreissenid mussel management or eradication in lakes is relatively undocumented in the freshwater management literature. This review presents information on 33 open water dreissenid mussel control projects in 23 North America lakes. We reviewed data from past dreissenid mussel control projects and identified patterns and knowledge gaps to help inform adaptive management strategies. The three key lessons learned include (1) pre- and post-treatment survey methods that are designed to meet management objectives are beneficial, e.g., by sampling for all life stages and taking into account that no survey method is completely comprehensive; (2) defining the treatment area—particularly ensuring it is sufficiently large to capture all life stages present—is critical to meeting management objectives; and (3) control projects provide an opportunity to collect water chemistry, effects on non-target organisms, and other efficacy-related data that can inform safe and effective adaptive management.

## Introduction

Invasive species pose a major threat to ecosystems worldwide and are associated with declines in biodiversity and ecosystem services^1,2^. Two particularly problematic aquatic invasive species (AIS) in North America are *Dreissena polymorpha* (zebra mussel; Pallas, 1771) and *D. bugensis* (quagga mussel; Andrusov, 1897). These dreissenid mussels are bivalves native to the Ponto-Caspian region in eastern Europe that were presumably introduced to the Great Lakes via ballast water from cargo ships in the mid- to late-1980s^3,4^. Secondary spread via recreational watercraft and related equipment accompanied by dreissenid mussels’ prolific reproduction and rapid growth rate have allowed dreissenid mussels to quickly establish in hundreds of waterbodies across North America^3,5–7^ (for the latest distribution maps see https://nas.er.usgs.gov/).

Once dreissenid mussels reach high densities in a waterbody, they can have a variety of impacts to the native ecosystem. Their filter feeding reduces concentrations of suspended solids and phytoplankton, consequently increasing light transmittance and submerged plant production^8^, as well as restructuring energy and nutrient fluxes, changing food web structure, and adversely affecting fish populations^7,9–12^. Additionally, dreissenid mussel biofouling has economic impacts to industry, including millions of dollars spent in mitigation by water treatment and power generation facilities^6,13,14^. These ecological and economic impacts have resulted in widespread efforts to curtail the species’ spread and impacts. Currently, prevention is the primary strategy for controlling the spread and impacts of dreissenid mussels in open waterbodies^15,16^; however, development and application of control strategies are increasing^17,18^.

Dreissenid mussel control efforts in open water include physical, biological, and chemical methods. Thus far, physical efforts have included manual removal by divers, creating anoxia with benthic mats, and desiccation from waterbody drawdowns^19–21^. Biological strategies have investigated the effectiveness of fish, crayfish, parasites, and microbes to reduce mussel populations by predation or infection^22–24^. For example, the biopesticide, Zequanox (Marrone BioInnovations, Davis, California), which incorporates a killed strain of *Pseudomonas fluorescens* (*Pf-*CL145A), a naturally occurring bacterium in soil and water, is registered by the U.S. Environmental Protection Agency (EPA) as a molluscicide for dreissenid mussel control. Although physical and biological methods have shown promise^19,25^, concern for non-target effects, efficacy, and/or cost have limited their adoption^26,27^.

Copper-based pesticides have a long history of use in aquatic ecosystem management and were used in open water to control dreissenid mussels as early as 2004^17,26,28–30^. Several forms of copper-based pesticides have been used in these efforts, including copper sulfate (CuSO_4_), Cutrine-Ultra (Applied Biochemists, Alpharetta, Georgia), Natrix (SePro Corporation, Carmel, Indiana), and EarthTec QZ (Earth Science Laboratories, Rogers, Arkansas). Currently, EarthTec QZ and Natrix are the only copper-based products registered by the U.S. EPA as molluscicides for dreissenid mussel control, although Cutrine-Ultra is registered for use as an algaecide, herbicide, and cyanobactericide.

Potassium chloride (KCl), or potash in its unrefined form, has also been used to control dreissenid mussels. KCl is not registered by the U.S. EPA as a molluscicide, but through site-specific regulatory exemption processes (i.e., Section 24 (c) Special Local Needs exemption^31^, Section 18 Emergency Exemption^32^) has been used in open water to control zebra mussels^33^. Section 24(c) of the Federal Insecticide, Fungicide, and Rodenticide Act (FIFRA) allows states to register a pesticide for a Special Local Need. Special Local Needs are defined as existing or imminent pest problems for which there is no appropriate federally registered pesticide available^31^. Alternatively, Section 18 of FIFRA authorizes the U.S. EPA to allow unregistered uses of pesticides to address emergency conditions^32^.

Although the currently available chemical products can effectively kill dreissenid mussels, they can have unintended negative effects on non-target organisms. For example, high concentrations of dissolved copper can be toxic to aquatic plants, algae, fish, snails, and other invertebrates^34–36^. KCl, at the concentrations lethal to dreissenids, has minimal effects on fish populations^26,37,38^, but can be lethal to shelled organisms, including native mollusks, crayfish, and zooplankton^39^.

Dreissenid mussel control projects have been ongoing since about 2004, yet the methods used and degree of reporting have varied greatly, hampering the development of best practices. Published reports of dreissenid mussel control projects, including Fernald and Watson^38^, Barbour et al.^40^, Lund et al.^33^, Hammond and Ferris^17^, and Luoma et al.^41^ provide important insight into site-specific strategies and outcomes. However, when reviewed singularly, the ability to identify patterns and fill knowledge gaps is limited.

We conducted a literature search and meta-analysis of open water dreissenid mussel control projects that have occurred in North America, including published and unpublished reports. Our goal was to identify knowledge gaps to inform future dreissenid management; to do that, we built a database using published and unpublished reports of both successful and failed dreissenid management projects. Where possible, we analyzed currently available data to inform lessons learned and offer recommendations for future management.

## Methods

We collected information on all reported open water dreissenid mussel control projects that involved the use of a molluscicide or pesticide (excluding drawdowns, manual removal, or in contained/industrial settings) in North America through direct contact to natural resource professionals as well as an exhaustive review of published literature. We define a ‘project’ for this review as a treatment that used the same molluscicide/pesticide within the same treatment area during a calendar year. However, if a lake was treated with different products (i.e., such as Zequanox and KCl) in the same year, this would be counted as two projects.

We contacted resource managers and researchers within the AIS community, including The Invasive Mussel Collaborative listserv (https://invasivemusselcollaborative.net), staff from manufacturers of registered dreissenid mussel control pesticide companies (i.e., Earth Science Labs, Marrone Bio Innovations, and ASI Group Ltd.), staff within agencies who are known to have conducted control projects (i.e., Minnesota Department of Natural Resources (MN DNR)), staff within agencies who are known to have conducted research for control (i.e., U.S. Geological Survey (USGS)), and others identified by the initial contacts. Each project was assigned a primary contact, herein referred to as the ‘project manager.’ The final list was shared with the Invasive Mussel Collaborative to confirm all projects were identified. We requested all available information on the project from the project manager, including summary reports, raw data, personal communication, and maps. Available data were organized in a narrative format and Microsoft Access database and categorized into pre-treatment, treatment, or post-treatment activities for further review (Table 1). When one project immediately followed a previous project (up to five weeks during non-reproductive winter months), activities that were considered post-treatment to the initial project were also considered pre-treatment for the subsequent project. For example, if part of a lake was treated but additional dreissenid mussels were found outside of the treatment area the following week, then a larger area was treated two weeks later, those surveys that occurred between the two treatments would be included as post-treatment for the first treatment and pre-treatment for the second treatment.

**Table 1:** Pre-treatment, treatment, and post-treatment details extracted from dreissenid mussel control projects.

Pre- and post-treatment survey details:	Treatment details:
Number of total surveys	Product(s)
Location and extent of surveys	Start and end dates
Time spent on surveys	Number and frequency of any subsequent bump treatments
Date(s) of surveys	Size of treated area
Methods and type(s) of surveys	Molluscicide target treatment concentration
Life stages of dreissenid mussels observed	Use of barriers
Environmental conditions	Observed maximum, minimum, and average product concentrations
Any other observations or notes	Project goal
	*Treatment depth* At or near the surface, at or near the bottom, or at multiple depths
	*Water chemistry and environmental measurements* Wind speed, wind direction, precipitation, water temperature, turbidity, dissolved oxygen, benthic dissolved oxygen, alkalinity, hardness, ammonia, total nitrogen, nitrate/nitrite levels, total phosphorus, potassium, chloride, biological oxygen demand, copper, carbonaceous oxygen demand, sodium, total suspended solids, magnesium, calcium, sulfate, dissolved organic carbon, conductivity, and pH
	*Impacts to non-target organisms* Chlorophyll *a* (phytoplankton productivity), phytoplankton, zooplankton, bacteria, benthic macroinvertebrates, native mussels, fish, and aquatic plants (macrophytes)

In addition to treatment logistical details, we extracted information on water chemistry and environmental measurements recorded during the project. Some measurements are important for determining proper application rates. For example, copper application can be informed by dissolved ion concentrations. Because copper bioavailability changes with water chemistry metrics (e.g., pH, dissolved calcium, dissolved magnesium)^42–44^, copper toxicity and effective treatment concentrations also change. Other environmental measurements, including dissolved oxygen and chlorophyll levels, provide insight into how conditions are changing for non-target organisms^26,34,42–46^.

We categorized each project’s goal as either rapid response eradication, established population eradication, suppression, or research. Rapid response eradication projects were defined as treatments of newly discovered infestations and were partial lake treatments conducted at the site of infestation within 6 months of discovery with the aim of eradication. Newly discovered infestations were lakes that had no previous known records of dreissenid mussel occurrences and the presumed introduction of dreissenid mussels was within the calendar year. Established population eradication projects, in contrast, were eradication attempts designed to treat the entire shoreline or surface area of a waterbody that had a known established population with ongoing active reproduction. Suppression projects worked to reduce but not eliminate dreissenid mussels from within the treated waterbody. Finally, research projects, in this context, included treatments that explicitly tested different methods or approaches, often with representative control sites. We acknowledge that some projects fit multiple definitions and that in some cases the management goal was unclear. Each project was classified with only one management goal, that most captured the breadth of the project. One project, Lake Minnetonka (St. Alban’s Bay), had goals as both a suppression and as a control project. For the purposes of this analysis, we treated that project as a suppression project because its size and scope contribute substantial insight into treating a site for population suppression.

To evaluate project outcomes, we reviewed available data to determine if dreissenid mussels were present within the treatment area in the short-term, outside the treatment area in the short-term, within the treatment area in the long-term, and outside the treatment area in the long-term. Short-term was defined as the period between the treatment and the first post-treatment monitoring event. If there were no post-monitoring events within the first year, we considered short-term results absent or null. Long-term included anything beyond the short-term period, and conclusions for long-term results were based on the more recent data. In projects where the treated area was the entire shoreline or surface area of the waterbody, all observations were considered “inside” treatment area, and no observations were for “outside” treatment area.

We wanted to assess the change over time of (1) the annual number of non-research control projects, (2) the size of rapid response and established population eradication project treated areas relative to the total surface area of the treated water body, and (3) the number of rapid response and established population eradication project pre-treatment survey methods for eradication projects. We conducted a visual verification of normality using Q-Q plots. For normally distributed data we conducted a Pearson correlation test, and for non-normally distributed data we conducted a Kendall rank correlation test. We interpreted correlation values as significant when p < 0.05.

Data were stored in a Microsoft Access database, and summary statistics and analyses were calculated and conducted using R software (R Development Core Team, version 3.6.2). All data are available at https://conservancy.umn.edu/handle/11299/231053. In addition, data were organized and visualized in an Esri ArcGIS StoryMaps web application which can be accessed at https://arcg.is/18frH50.

## Results

We identified 33 open water dreissenid mussel control projects in 23 lakes across North America from 2004 to 2021 (Table 2). Projects were categorized as rapid response eradication (n = 16 projects), established population eradication (n = 8 projects), suppression (n = 3 projects), or research (n = 6 projects). The summarized narrative for each project and all available data are publicly available in the University of Minnesota’s Data Repository (https://conservancy.umn.edu/handle/11299/231053). Additionally, narratives describing each project as well as data visualizations are available in an Esri ArcGIS StoryMaps web application (https://arcg.is/18frH50). Presenting the data this way communicates the nuances and details of these projects that would otherwise be lost in a table and makes the data more readily accessible to a broad audience.

**Table 2:** Details for control projects in different lakes across North America.

Lake name (state/province)	Goal^1^	Pre-treatment monitoring^2^	Treatment								Post-treatment monitoring^2^	Outcomes: Dreissenid mussel presence^5^			
			Year	Product	Treatment area (ha) (% of lake)	Treatment duration (d)	Treatment depth	Barrier use (Y/N)	Non-target monitoring^3^	Environmental/water chemistry^4^		ST/IT	LT/IT	ST/OT	LT/OT
Base Lake (NE; Offutt Air Force Base)	EPE	DS, Vel	2008	CuSO4	46.54 (100%)	1.25	Surface	N	Fish	Alk, Cu, pH, Temp	Plate, Vel	N	Y	NA	NA
	EPE	–	2009	CuSO4	46.54 (100%)	1.25	Surface	N	Fish	Alk, Cu, pH, Temp	Plate, Vel, Wade	N	Y	NA	NA
Billmeyer Quarry (PA)	EPE	DS	2017	EarthTec QZ	5.99 (50%)	37	Multiple	N	Fish, Phyto, Zoop	Alk, Cu, DO, Hard, Temp	eDNA, Vel, Wade	N	N	NA	NA
Bone Lake (MN)	RRE	DS, Snork, Vel, Wade	2019	EarthTec QZ	0.23 (0.26%)	10	–	Y	–	Cu, Prec, Temp, WD, WS	Plate, Vel, Wade	N	Y	Y	Y
Christmas Lake (MN)	RRE	DS, Snork, Vel, Wade	2014	Zequanox	0.03 (0.03%)	11	Surface	Y	–	DO, Temp, Turb	DS, Snork	N	Y	Y	Y
	RRE	DS, Snork	2014	EarthTec QZ	0.30 (0.28%)	10	Surface	Y^6^	–	Cu, Temp	–	N	Y	Y	Y
	RRE	–	2014	KCl	0.30 (0.28%)	1	Surface	Y	–	Cl, Cond, K, Temp	DS, Snork, Wade	N	Y	Y	Y
	RRE	DS, Snork, Wade	2015	KCl	0.30 (0.28%)	10	Surface	Y	–	Cl, Cond, K, Temp	–	N	Y	Y	Y
	RRE	–	2015	KCl	4.45 (4.12%)	12	Surface	Y	–	Cl, Cond, K, Temp	DS, Equip, Plate, Snork, Vel, Wade	N	Y	Y	Y
Crosley Lake (IN)	EPE	–	2016	EarthTec QZ	0.61 (50%)	14	Surface	N	–	Cu	Vel, Wade	N	N	NA	NA
Deep Quarry Lake (IL)	R	Cage, Snork, Wade	2012	Zequanox	0.01 (0.04%)	1	Bottom	Y	Chl	BOD, NH3, Nit, TN, TP, Turb	Cage, UWA, Wood	Y	Y	Y	Y
	R	Cage, Vel	2013	Zequanox	0.03 (0.2%)	1	Bottom	Y	–	Cond, DO, pH, Temp, Turb	Cage, UWA, Vel	Y	Y	Y	Y
Lake Erie (MI)	R	–	2014	Zequanox	0.08 (0.00%)	1	Bottom	Y	–	Cond, DO, pH, Temp, Turb	–	Y	Y	Y	Y
Highland Lake (IL)	EPE	Plate, Vel	2020	EarthTec QZ	15.78 (38%)	30	Surface	N	Zoop	Cu, Temp	Plate, Vel	Y	Y	NA	NA
	EPE	Plate, Vel	2021	EarthTec QZ	15.78 (38%)	30	Surface	N	Zoop	Cu, Temp	Plate, Vel	Y	Y	NA	NA
Lake Independence (MN)	RRE	Wade	2014	EarthTec QZ	0.20 (0.06%)	7	Surface	Y	–	Cu	Vel^7^, DS, Snork, Wade	N	Y	Y	Y
	RRE	DS, Snork, Wade	2015	KCl	0.30 (0.09%)	42	–	Y	–	K, Temp	DS, Plate, Snork	N	Y	Y	Y
Lake Irene (MN)	RRE	DS, Equip, Wade	2011	Cutrine Ultra	4.05 (1.56%)	–	–	Y	–	Cu	DS, Vel	N	Y	Y	Y
Lake Marion (MN)	RRE	DS, Snork, Wade, Vel	2017	EarthTec QZ	2.43 (1.13%)	8	Surface	Y	–	Alk, Cond, Cu, DO, Hard, pH, Temp	DS, Snork, Vel	Y	Y	Y	Y
Lake Michigan (Good Harbor Bay) (MI)	R	Quad	2019	Zequanox	0.03 (0.00%)	1	Bottom	Y	Bac, BI, Fish	BDO, COD, Cond, DO, DP, NH3, pH, Temp, TP, TSS, Turb	Quad	Y	Y	Y	Y
Millbrook Quarry (VA)	EPE	Cage, DS, Wade	2006	KCl	4.86 (100%)	18	Surface	N	–	K	Cage, DS, UWA, Video	N	N	NA	NA
Lake Minnetonka (Robinson Bay) (MN)	R	Cage	2014	Zequanox	0.01 (0.00%)	3	Multiple	Y	–	Alk, Cond, DO, Hard, NH3, pH, Temp, TN, TP, WD, WS	Cage, Plate	Y	Y	Y	Y
Lake Minnetonka (St. Alban's Bay) (MN)	S	Cage, DS, Vel	2019	EarthTec QZ	66.30 (1.13%)	10	Surface	N	BI, Chl, Fish, NM, Zoop	Alk, Ca, Cl, Cond, Cu, DO, DOC, Hard, K, Mg, Na, pH, SO4, Temp	Cage, DS, Plate, Vel	N	Y	Y	Y
Lake Minnewashta (MN)	RRE	Snork, Vel, Wade	2016	EarthTec QZ	11.74 (4.27%)	10	Surface	Y	AP, Fish, NM	Cond, Cu, DO, pH, Temp	Equip	N	Y	Y	Y
	RRE	DS, Plate, Snork, Vel, Wade	2017	EarthTec QZ	0.40 (0.15%)	9	Surface	Y	AP, Fish	Cond, Cu, DO, pH, Temp	DS, eDNA, Equip, Rake, Snork, Vel, Wade	N	Y	Y	Y
Lake Ossawinamakee (MN)	S	DS, Rake, Vel, Wade	2004	Cutrine Ultra	10.52 (3.66%)	–	Surface	N	BI	Cu	–	N	Y	Y	Y
	S	–	2005	Cutrine Ultra	10.52 (3.66%)	–	Surface	N	BI	Cu	–	N	Y	Y	Y
Richland Chambers Reservoir (TX)	RRE	DS	2018	EarthTec QZ	2.02 (0.01%)	8	Multiple	Y	–	Cu, Temp	–	N	N	–	–
Rose Lake (MN)	RRE	DS, Equip, Rake, Wade	2011	Cutrine Ultra	4.05 (0.83%)	15	–	Y	–	Cu	DS, Equip, Vel, Wade	N	Y	Y	Y
Round Lake (MI)	R	Cage, NMI, quad	2017	Zequanox	9.00 (6.67%)	1	Bottom	N	BI, NM, Peri	Alk, Cond, DO, Hard, pH, Temp, Turb	Cage, NMI, quad	Y	Y	Y	Y
Ruth Lake (MN)	RRE	DS, Vel, Wade	2015	EarthTec QZ	1.17 (0.48%)	8	–	Y		Cu, Temp	DS, Plate, Snork, Vel	N	Y	Y	Y
Valley Lo Lake (IL)	EPE	Vel, Wade	2021	EarthTec QZ	5.67 (50%)	1	Bottom	N	Zoop	Alk, Ca, Cl, Cu, DO, DOC, K, Mg, Na, pH, SO4, Temp	Vel, Wade	N	N	NA	NA
Lake Winnipeg (MB)	RRE	Equip	2014	KCl	–	19	Surface	Y	–	K, Temp	–	N	Y	Y	Y

For full details and descriptive notes, see https://conservancy.umn.edu/handle/11299/231053. Outcomes report the presence of dreissenid mussels (Y = present, N = not present).

^1^Project goals: RRE = rapid response eradication, EPE = established population eradication, S = suppression, R = research.

^2^Pre- and post-treatment surveying types include Cage = monitoring dreissenid mussels held in cages or chambers in the waterbody, DS = diving survey, eDNA = eDNA sampling, Equip = equipment inspection, NMI = assessment of native mussel infestation (i.e., density or number), Plate = settlement plate use, Quad = dreissenid density at a given location (i.e., plot or quadrat), Rake = inspection of removed vegetation (i.e., by rake toss), Snork = snorkeling survey, UWA = assessment of underwater attachment to substrates and macrophytes, Vel = veliger tow, Video = underwater video inspection, Wade = wading survey, Wood = measuring attachment to submerged woody surfaces (i.e., density, total number).

^3^Non-target monitoring types include monitoring of: AP = aquatic plants (macrophytes), Bac = bacteria, BI = benthic invertebrates, Chl = chlorophyll, Fish = fish, NM = native mussels, Peri = periphyton, Phyto = phytoplankton, Zoop = zooplankton.

^4^Environmental and water chemistry metrics include: Alk = alkalinity, NH3 = ammonia, BDO = benthic dissolved oxygen, BOD = biological oxygen demand, Ca = calcium, COD = carbonaceous oxygen demand, Cl = chloride, Cond = conductivity, Cu = copper, DOC = dissolved organic carbon, DO = dissolved oxygen, DP = dissolved phosphorus, Hard = hardness, Mg = magnesium, Nit = nitrate or nitrite, pH = pH, K = potassium, Prec = precipitation, Na = sodium, SO4 = sulfate, TN = total nitrogen, TP = total phosphorus, TSS = total suspended solids, Turb = turbidity, Temp = water temperature, WD = wind direction, WS = wind speed.

^5^Outcomes refers to: ST = short-term (before post-treatment sampling or within one year), LT = long-term (after post-treatment sampling or after one year), IT = within treated area, OT = outside of treated area; Y = dreissenid mussels present, N = dreissenid mussels not present, U = unknown; projects that treated 100% of the waterbody, or 100% of the shoreline do not have OT responses.

^6^Initially no barrier was installed; copper concentrations decreased from 1 to 0.5 mg/L Cu within 8 h, prompting installation of a floating barrier.

^7^Veliger tows taken during the treatment; came back with no veligers present.

**Figure 1:**
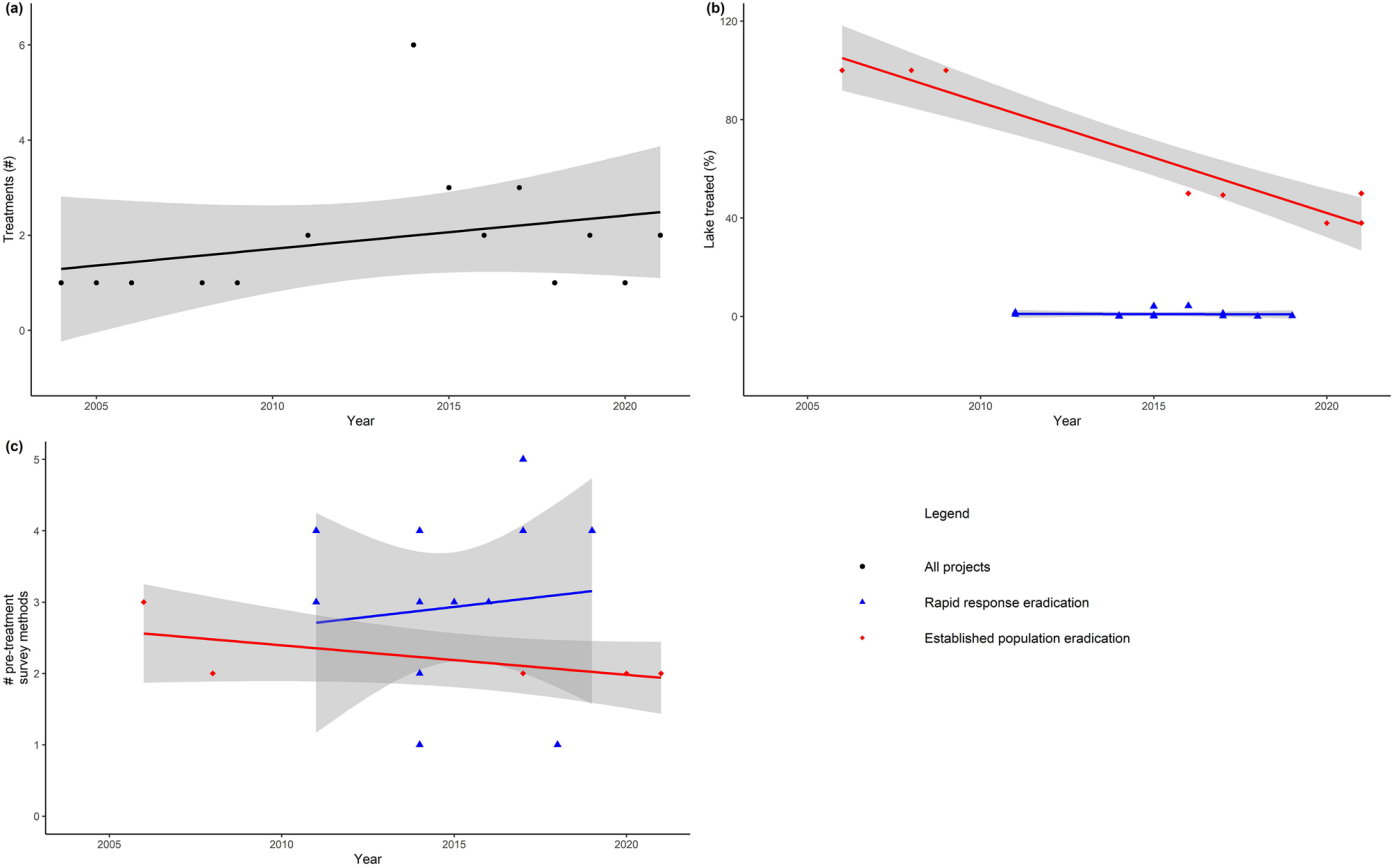
(**a**) Total number of rapid response eradication, established population eradication, and suppression projects per year. (**b**) The percent of a lake’s surface area treated for each rapid response eradication and established population control project. (**c**) Number of pre-treatment survey methods conducted per rapid response eradication, established population eradication, and suppression control project.

Since 2004, the annual number of dreissenid control projects has not changed (*r* = 0.27, *p* = 0.21, Fig. 1a). During the first half of the treatment time range (2004–2012), eight projects were conducted (2 RRE, 3 EPE, 2 S, 1 R). During the second half of the treatment time range (2013–2021), 25 projects were conducted (14 RRE, 5 EPE, 1 S, 5 R). The size of the treated areas, relative to the total surface area of the lake, has not changed for rapid response eradication projects (*r*_*τ*_ = -0.10, *p* = 0.61, Fig. 1b), but has decreased for established population eradication projects (*r* = -0.96, *p* < 0.01, Fig. 1b) as those projects have shifted from entire surface area treatment to entire shoreline treatment. The number of pre-treatment survey methods per project has not changed for either rapid response or established population eradication projects (i.e., more recent projects do not use more or fewer pre-treatment survey methods; *r* = 0.10, *p* = 0.60, and *r*_*τ*_ = -0.60, *p* = 0.14, respectively; Fig. 1c). For non-research projects, the number of pre-treatment surveys is unrelated to the treatment area (i.e., conducting more pre-treatment surveys has not resulted in larger or smaller treatment areas; *r*_*τ*_ = –0.19, *p* = 0.28).

Pre- and post-treatment practices were similar across all projects (Table 3). Across all projects examined here, project managers used an average of 3.0 post-survey methods (n = 27 projects), slightly more than the average of 2.6 survey methods used pre-treatment (n = 26). Post-treatment monitoring added 1.23 ± 1.41 [mean ± standard deviation (sd)] practices that were not conducted during pre-treatment. Simultaneously, post-treatment practices omitted 0.94 ± 1.09 (mean ± sd) practices that had been conducted pre-treatment.

**Table 3:** Pre- and post-treatment practices reported for 33 dreissenid mussel control projects across North America, combined and separated by project type.

	All projects		Breakdown by project type							
			Rapid response eradication		Established population eradication		Suppression		Research	
	Pre	Post	Pre	Post	Pre	Post	Pre	Post	Pre	Post
Total number of projects with reported survey methods during pre- or post-treatment periods (*n*)	26	27	14	12	6	8	2	1	5	5
Average number of survey methods	3	3	3	4	2	3	4	–	2	2
Breakdown by survey method: *n* (%)										
Diving searches	16 (62)	12 (44)	11 (79)	10 (83)	3 (50)	1 (13)	2 (100)	1 (100)	–	–
Wading or shoreline surveys	16 (62)	10 (37)	11 (79)	8 (67)	3 (50)	4 (50)	1 (50)	–	1 (20)	–
Veliger tows	13 (50)	17 (63)	6 (43)	8 (67)	4 (67)	7 (88)	2 (100)	1 (100)	1 (20)	1 (20)
Snorkeling surveys	9 (35)	8 (30)	8 (57)	8 (67)	–	–	–	–	1 (20)	–
Mortality assessment using caged dreissenid mussels	6 (23)	6 (22)	–	–	1 (17)	1 (13)	1 (50)	1 (100)	4 (80)	4 (80)
Equipment inspections	3 (12)	4 (15)	3 (21)	4 (33)	–	–	–	–	–	–
Settlement plates	3 (12)	10 (37)	1 (7)	4 (33)	2 (33)	4 (50)	–	1 (100)	–	1 (20)
Inspection of removed vegetation via rake tosses and other methods	2 (8)	1 (4)	1 (7)	1 (8)	–	–	1 (50)	–	–	–
Monitoring dreissenid density in quadrats or at sites	2 (8)	2 (7)	–	–	–	–	–	–	2 (40)	2 (40)
Measuring native mussel infestation by dreissenid mussels	1 (4)	1 (4)	–	–	–	–	–	–	1 (20)	1 (20)
Quantifying dreissenid mussels attached under water to substrate and vegetation	–	3 (11)	–	–	–	1 (13)	–	–	–	2 (40)
Environmental DNA analysis	–	2 (7)	–	1 (8)	–	1 (13)	–	–	–	–
Underwater video inspection	–	1 (4)	–	–	–	1 (13)	–	–	–	
Quantifying dreissenid attachment to submerged woody material	–	1 (4)	–	–	–		–	–	–	1 (20)

During treatment, environmental parameters were often not well-monitored. Project managers were most likely to measure water temperature (76% of projects, n = 25), dissolved oxygen (36%, n = 12), conductivity (36%, n = 12), and pH (36%, n = 12); however, other data (see Table 1) were unavailable in up to 76–97% of projects (for example, 76% or n = 25 projects did not include potassium and 97% or n = 32 projects did not include dissolved phosphorus; see Table 2, and for further discussion see the upcoming section “Lesson 3”). Of 20 projects that used copper-based products (EarthTec QZ, Cutrine Ultra, CuSO_4_); of those, only 10% (n = 2) reported the metrics necessary to help predict site-specific copper toxicity.

Information on non-target effects during or after treatment was limited; only two rapid response eradication projects (12.5% of projects in that category), six established population eradication projects (75%), three suppression projects (100%), and three research projects (50%) reported at least one non-target observation. The mean number of non-target organism groups assessed per project were 2.5, 1.3, 2.3, and 2.3 for rapid response eradication, established population eradication, suppression, and research projects, respectively (see Table 1 for a list of non-target organism groups). Of the non-target assessments, fish were the most common non-target organism group observed (21% of projects, n = 7), followed by zooplankton (15%, n = 5), benthic invertebrates (15%, n = 5), and native mussels (9%, n = 3).

**Figure 2:**
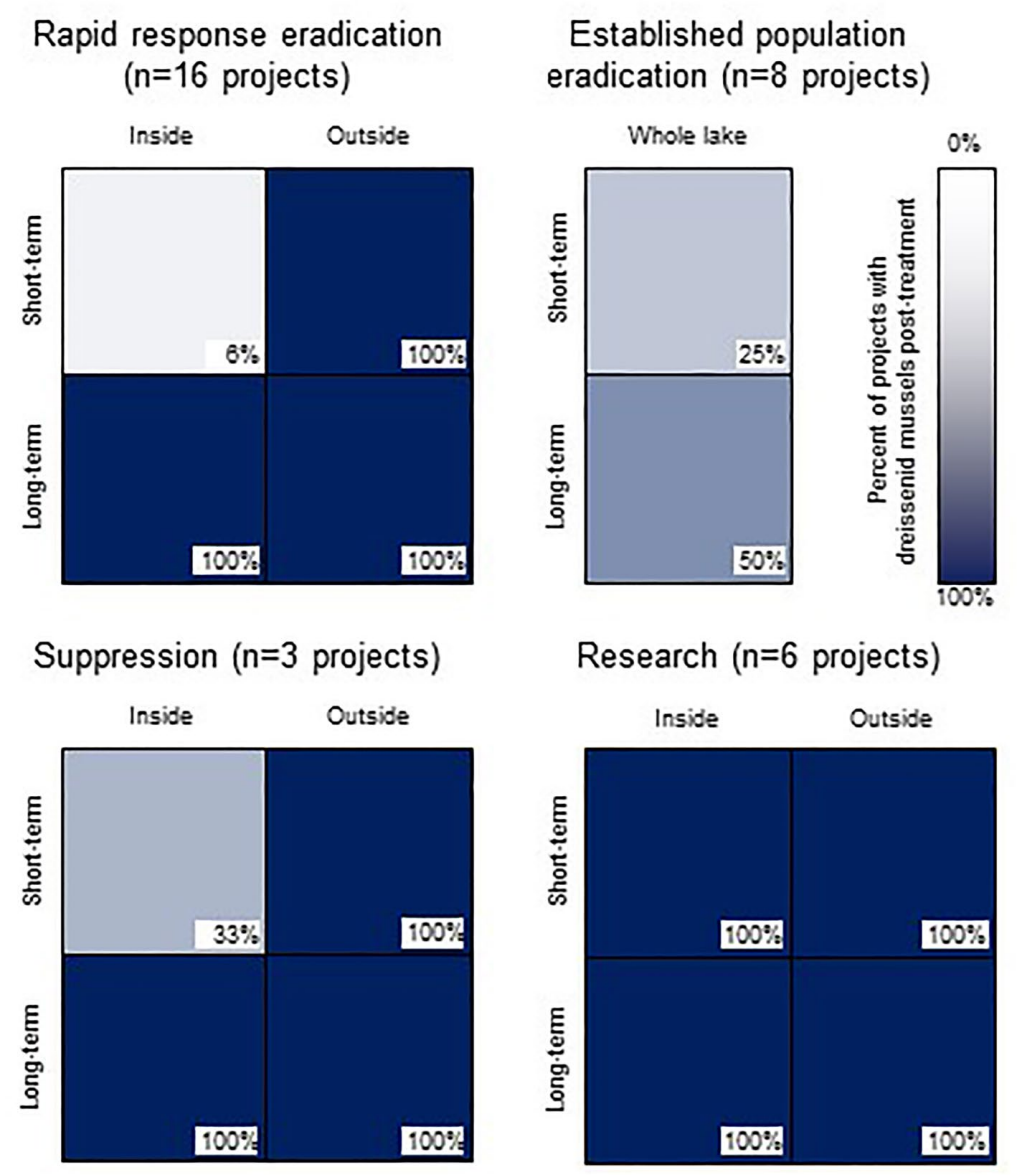
Dreissenid mussel control project outcomes for different management objectives—lighter colors indicate more success. Results are for inside and outside the treated area, in the short-term (within one year or before a post-treatment survey, whichever comes first) and long-term. Due to limited outcome data, Richland Chambers Reservoir has been excluded.

Rapid response eradication projects (n = 16 projects) were generally successful at eradicating dreissenid mussels within the treated area in the short-term (94% of lakes, n = 15 lakes; Fig. 2). However, in all cases where data were available (100% of lakes with treatments, n = 15 lakes; one lake did not have long-term data) dreissenid mussels were ultimately discovered outside the treated area and eradication was unsuccessful. In Lake Minnewashta, project managers speculate that the population observed post-treatment was the result of a reintroduction, not a failed project (Keegan Lund, Minnesota Department of Natural Resources, written communication, 2022). Although not considered a success by our criteria (dreissenid mussel presence 1 + year post-treatment), two rapid response eradication projects have shown promising results. Ruth Lake (treated in 2015) and Bone Lake (treated in 2019) both had confirmed zebra mussel presence either through veliger presence (Bone Lake) or the presence of one adult (Ruth Lake) but have had no reported adult zebra mussel observations since.

Established population eradication projects (n = 8 projects) were similarly successful at short-term eradication of dreissenid mussels in the treated area (75%, n = 6) and were more successful in long-term eradication throughout the waterbody (50% of projects, n = 4; Fig. 2). Only four lakes had long-term eradication of an entire waterbody (Valley Lo Lake, Billmeyer Quarry, Crosley Lake, and Millbrook Quarry).

Suppression projects (n = 3 projects) reduced or eradicated dreissenid mussels within the treatment area in the short-term, as intended (Fig. 2). However, the treated areas were small relative to the size of the entire lake and as expected, within 1–2 years the treated areas were reinfested from other areas within the same lake (St. Albans Bay within Lake Minnetonka and Lake Ossawinamakee).

Research-focused projects (n = 6 projects) generally did not eliminate dreissenid mussels inside or outside the treated areas either in the short-term or long-term (Lake Erie, Good Harbor Bay, Deep Quarry Lake, Round Lake, Robinson Bay in Lake Minnetonka) (Fig. 2). Instead, those projects were experiments designed to refine application methods, with an emphasis on technique rather than control, or to better understand non-target and/or ecosystem responses to control methods. For example, these projects demonstrate advantages and disadvantages of targeting the application of product to benthic, surface water, or the whole water column; effectiveness of different products applied within small enclosures; and effectiveness of varying concentrations in natural lake water.

## Discussion

Although we have organized a large dataset for 33 dreissenid mussel control projects, inconsistent data availability between each project prevented thorough quantitative comparisons. Despite this, we believe that the data available offer the opportunity to identify several important trends and insights to inform future dreissenid control projects. Although there are more lessons to be learned from these data, we highlight three major themes below that can inform safe and effective adaptive management, demonstrate important gaps in current knowledge, and emphasize key features of the most successful outcomes.

### Lesson 1: Pre- and post-treatment survey methods that are designed to meet management objectives are beneficial

For any treatment, project managers can begin by defining their treatment goals, objectives, and threshold for acceptable effects to non-target organisms. Each situation will have a different cost to benefit ratio for dreissenid control. Once a project’s goals are defined, project managers can identify their treatment area and treatment methods.

Determining a treatment area, however, can be challenging because low densities of dreissenid mussels can be difficult to detect making it difficult to define the extent of their infestation. For example, dreissenid mussels are small, only reaching average maximum adult sizes of 23 mm (*Dreissena polymorpha*) and 33 mm (*D. bugensis*)^47^, and water conditions such as the abundance of submersed aquatic vegetation or high turbidity can make those adults challenging to locate through underwater search methods^33^. Furthermore, veligers are often present at low densities and only detectable with a microscope^3^. One useful early detection tool is monitoring for eDNA^48,49^; however, the application of eDNA detection is not yet reliable for defining the spatial extent, scale, or stage of an infestation^50,51^.

To account for detection challenges, project managers can carefully consider appropriate combination of pre-treatment survey methods that will be most likely to accurately determine the extent of an infestation and thus inform a treatment area. Infestation areas can be estimated using a variety of survey methods (e.g., diving searches and veliger tows), although each method has different advantages and disadvantages^33,52–54^. For example, even skilled divers will fail to detect all dreissenid mussels^54^, and multiple plankton tows may be needed to detect veligers at low-density. Even with well-defined survey methods, the difficulties in exhaustively detecting dreissenid mussels in freshwater systems are many and this limitation warrants consideration when setting the project objectives and defining treatment areas.

Further, project managers can delineate the populations of both adult and pre-settled dreissenid mussel life stages. Because adult and pre-settled life stages have different habits (e.g., planktonic vs. attached) and can be found in different locations (e.g., in the water column vs. on the lakebed), treatment methods may vary when addressing one or both life stages. Among the projects examined, project managers who conducted rapid response projects to eradicate dreissenid mussels from a partial waterbody containing both adults and veligers were never successful (i.e., Bone Lake, Lake Marion). In the case of Bone Lake, veliger tows were conducted prior to treatment, but the treatment was initiated before the veliger tows were analyzed. Project managers later learned that veligers were present outside the treatment area, resulting in a presumed failed eradication attempt. In Lake Marion, project managers found low veliger density inside and outside of the defined treatment area before treatment but selected and treated an area based on the location of adults only, and post-treatment sampling found live veligers in the treatment area. In both cases, pre-treatment surveys for veligers followed by appropriately accounting for veligers in treatment plans may have led to a different treatment strategy such as a lake-wide treatment or no further control efforts. It is also important to note that timely treatment is needed for rapid response projects to effectively control new infestations prior to reproductive establishment. As demonstrated in these data, rapid response efforts have had minimal success because while effective within the treatment area, mussels were later discovered outside, indicating that the treatment area was not large enough and that better survey methods would help.

In contrast, project managers that potentially accounted for detection uncertainty and then treated for all possible life stages as part of entire population eradication projects were more likely to meet their management objectives (e.g., Billmeyer Quarry, Valley Lo Lake). Billmeyer Quarry had been infested for at least 12 years when it was treated and was known to have an established and reproducing dreissenid mussel population. Project managers targeted all life stages of this population with a treatment along the entire perimeter of the lake covering a total of 50% of the waterbody’s surface area. No live veliger or adult mussels have been detected in tows and eDNA samples in the 4 years since 2017, indicating successful eradication. Valley Lo Lake was also confirmed to contain veligers and was treated in a similar manner to Billmeyer Quarry, including 50% of the surface area along the lake perimeter. Within one week, veliger concentrations were reduced by 95%, and within one month, no live veligers were detected. Caged adult zebra mussels held and monitored within the lake at the surface and the bottom experienced 100% mortality after 3 and 10 days, respectively (David Hammond, Earth Science Laboratories, Inc, written communication, 2022).

While pre-treatment methods are important for defining treatment areas, post-treatment methods are key for evaluating the success of the project. As with pre-treatment survey methods, post-treatment survey methods that accurately assess both adult and veliger mussel presence would be helpful. Project managers may want to consider using the same survey methods post-treatment that were used pre-treatment. This standardization allows project managers to evaluate treatment effectiveness, and may be particularly important for suppression projects, where the project goal is more nuanced than dreissenid mussel presence/absence (see Lake Minnetonka, St. Alban’s Bay as an example).

Project managers may want to include additional post-treatment survey methods for all projects, especially rapid response eradication projects. Rapid response eradication projects typically require fast decisions and management actions; after treatment, the project urgency diminishes, and project managers can use slower survey methods such as settlement plates or end-of-season equipment inspections to make treatment evaluation more robust.

Finally, the following recommended for partial lake treatments. Underwater search efforts that include dive surveys that focus search efforts at the infestation site would be most beneficial. Additional dive surveys could be conducted at other potential introduction sites on that waterbody (e.g., marinas, private boat access sites). Those diving searches may be bolstered by wading or snorkeling surveys and should be coupled with veliger tows and eDNA sampling. Veliger tows and eDNA sampling could be conducted both near the infestation as well as at additional sites that are distinct from the infestation site to detect veligers and determine if reproduction is ongoing. Additionally, using standardized survey methods pre- and post-treatment would be helpful. One exception is rapid response eradication projects, where a method like settlement plates is too time-intensive to be practical pre-treatment, yet is valuable as a post-treatment measure, increasing confidence in the evaluation of project success.

### Lesson 2: Defining the treatment area is critical to meeting management objectives

Regardless of which pre- and post-treatment survey methods are used, achieving management goals will be dependent on how well the treatment area is defined. If the management goal is eradication, the treatment area would need to include all reproducing parts of the population. If the management goal is suppression, managers may need to be very specific in their expectations for how different parts of the population will change in different parts of the waterbody.

Among the projects we examined, nearly all non-research projects effectively eliminated dreissenid mussels within the treatment area in the short term. However, rapid response eradication treatments often fell short when additional dreissenid mussels were found outside the treatment area following treatment. When this happens, project managers would need to decide whether to apply additional treatments to a larger area (e.g., Christmas Lake, Lake Irene) or suspend efforts altogether (e.g., Bone Lake). In Christmas Lake, project managers initially treated a small area, then within days found additional zebra mussels just outside that treated area. The treatment area was increased three times and was treated on five occasions over two years with three different molluscicides^33^. In the end, zebra mussels were found outside of the largest treated area, additional treatments were suspended, and the lake remains designated as an infested waterbody with zebra mussels present lake wide. This indicates that although treatments were effective within the treatment areas, the reason some projects did not meet their management goals was because their treatment areas may not have been as well defined.

One commonality among projects that eradicated dreissenid mussels in a whole waterbody was that treatment was applied to large areas that constitute a ‘whole lake’ application as part of established population eradication projects. This observation, as compared to rapid response eradication projects, highlights the benefits of adequately defining the spatial extent of an infestation area so project managers can better determine the necessary treatment area. Furthermore, accounting for the uncertainty of dreissenid mussel detection efforts is warranted due to the inherent limitations^47^ of locating and isolating all dreissenid mussel populations in lakes^55^. Limiting the treatment to an area immediately around known mussel locations may fail to capture an entire population, especially planktonic veligers, resulting in a need for additional treatments or a failed eradication attempt.

The failure of past rapid response eradication projects to adequately define the area of infestation may be the result of surveys that are limited in temporal and spatial coverage (see Lesson 1) and/or a desire to reduce treatment costs and environmental effects, in turn leading to dose rates that ultimately proved sublethal. Project managers may want to consider a generous definition of the infested area, or where appropriate and/or feasible, consider entire shoreline or surface area treatments. We acknowledge that this may result in treating areas that are not yet invaded, but, in the case of projects where the end goal is eradication, the costs of a failed project (e.g., time, money, ecological effects) would need to be evaluated versus the costs of a one-time over-treatment.

### Lesson 3: Control projects provide an opportunity to collect data that can inform safe and effective adaptive management

Adaptive management is the process of learning from past experiences and modifying future actions in response to new information. This can be a highly effective approach when it includes information from many sources^56,57^. However, robust data on water chemistry, environmental characteristics, non-target effects, and positive ecosystem responses before, during, or after dreissenid mussel control projects are frequently not collected, making the retrospective evaluation of project outcomes difficult or impossible. Collecting and sharing standardized data could improve future project practices and success rates.

Water chemistry and environmental conditions can be essential for determining appropriate product application. Although all the projects we reviewed had measured some aspect of water or environmental conditions, not all measured the metrics necessary for developing an adaptive treatment regime (see Table 4). For example, toxicities of copper-based products are affected by competing ion concentrations in the water, as well as by environmental characteristics like dissolved organic carbon and water temperature^26,42,58–61^. If these water chemistry variables are measured, they can be used in bioavailability models (e.g., the Biotic Ligand Model, Visual MINTEQ, and others) to predict the appropriate concentration of copper needed to achieve a desired toxicity response [e.g., a concentration that kills 50% of a species (LC50)]. This predictive capability allows project managers to minimize non-target effects and cost while achieving effective control.

**Table 4:** Water chemistry and environmental characteristics required to prescribe appropriate concentrations of different pesticides for dreissenid control.

	Zequanox®	Potassium chloride (KCl) or potash	Copper-based pesticides (i.e., copper sulfate, Cutrine®-Ultra, Natrix™, EarthTec QZ®)
Alkalinity			++^42, 58–61^
Ca			++^42, 58–61^
Cl		+^65^	++^42, 58–61^
Cu			+^42, 58–61^
Dissolved organic carbon (DOC)			+^42, 58–61^
Hardness			+^42, 58–61^
Humic acid (HA)			++^42, 58–61^
K		+^65, 66^	++^42, 58–61^
Mg			++^42, 58–61^
Na		+^65, 66^	++^42, 58–61^
pH		++^65, 66^	+^42, 58–61^
S			++^42, 58–61^
Salinity		++^65, 66^	
SO_4_			++^42, 58–61^
Specific conductivity		+^65, 66^	
Total dissolved solids		++^65, 66^	
Turbidity or spectroscopy	+^25, 45, 67, 68^		
Water temperature	+^25, 45, 67, 68^	+^65, 66^	+^26, 42, 58–61^

+, indicates minimum requirements; ++, indicates most complete requirements for more accurate assessment.

Chemical products used to control dreissenid mussels can have unintended effects on non-target organisms^37, 62^; however these effects have rarely been monitored. Knowing which non-target organisms are present and how they respond to treatments can help project managers ensure treatments are not causing harm to non-target organisms^27, 36–38^. Indeed, non-target effects are an important concern for project managers^63^. In our review, only 42% of projects (n = 14 projects) included any information on non-target effects (Table 2). Conversely, collecting data that may demonstrate the environmental benefits of control efforts are also warranted so that managers can more fully assess the benefits of attempting eradication versus doing nothing^64^. Quantifying the tradeoffs between non-target effects and ecosystem benefits of dreissenid mussel control projects, in particular those that aim for established population eradication or suppression strategies, will better inform safe and effective adaptive management.

## Future directions

In this paper, we identify patterns and knowledge gaps to inform adaptive management strategies based on the largest review and meta-analysis, to the best of our knowledge, of a comprehensive dataset for dreissenid mussel control projects. Although these lessons are instructive, they are not inclusive and we expect additional lessons could be learned as new questions are asked, gaps are filled, or future control project data become available. It will be useful to maintain and update this database in a centralized and publicly available location to ensure that managers and researchers can most effectively and efficiently learn lessons from ongoing control efforts (i.e., https://arcg.is/18frH50, https://invasivemusselcollaborative.net/research-and-projects/toxicity-testing/, and https://www.dnr.state.mn.us/invasives/aquaticanimals/zebramussel/pilot_project.html). To that end, data collected as part of future control projects ideally would be standardized, robust, and publicly available. This would facilitate more quantitative analyses and comparisons to identify trends over time. For example, questions that these data could address include optimal timing of treatment, effects of water temperatures and chemistries, and efficacy of the frequency and timing of multiple applications within a project. Improved data collection on non-target effects and ecosystem benefits would also allow us to better assess the tradeoffs of control efforts.

## Data Availability

All data and codes are available at https://conservancy.umn.edu/handle/11299/231053. In addition, data were organized and visualized in an Esri ArcGIS StoryMaps web application which can be accessed at: https://arcg.is/18frH50.

## References

[1] Ehrenfeld JG (2010). Ecosystem consequences of biological invasions. Annu. Rev. Ecol. Evol. Syst..

[2] Walsh JR, Carpenter SR, Van Der Zanden MJ (2016). Invasive species triggers a massive loss of ecosystem services through a trophic cascade. Proc. Natl. Acad. Sci. USA.

[3] Mackie GL (1991). Biology of the exotic zebra mussel, *Dreissena polymorpha*, in relation to native bivalves and its potential impact in Lake St. Clair. Hydrobiologia.

[4] Mills EL (1996). A review of the biology and ecology of the quagga mussel (*Dreissena bugensis*), a second species of freshwater dreissenid introduced to North America. Am. Zool..

[5] Mills EL (1993). Colonization, ecology, and population structure of the ″Quagga″ mussel (Bivalvia: Dreissenidae) in the lower Great Lakes. Can. J. Fish. Aquat. Sci..

[6] Strayer DL (2009). Twenty years of zebra mussels: Lessons from the mollusk that made headlines. Front. Ecol. Environ..

[7] Higgins SN, Vander Zanden MJ (2010). What a difference a species makes: a meta-analysis of dreissenid mussel impacts on freshwater ecosystems. Ecol. Monogr..

[8] MacIsaac H (1996). Potential abiotic and biotic impacts of zebra mussels on the inland waters of North America. Am. Zool..

[9] McEachran MC (2018). Stable isotopes indicate that zebra mussels (*Dreissena polymorpha*) increase dependence of lake food webs on littoral energy sources. Freshw. Biol..

[10] Hansen GJA (2020). Walleye growth declines following zebra mussel and *Bythotrephes* invasion. Biol. Invasions.

[11] Zorn TG, Kramer DR (2021). Changes in habitat conditions, fish populations, and the fishery in northern Green Bay, Lake Michigan, 1989–2019. N. Am. J. Fish Manag..

[12] Depinto, J. Understanding declining productivity in the offshore regions of the Great Lakes. 1–73. https://policycommons.net/artifacts/2129008/understanding-declining-productivity-in-the-offshore-regions-of-the-great-lakes/2884307/ (2020).

[13] Connelly NA, O’Neill CR, Knuth BA, Brown TL (2007). Economic impacts of zebra mussels on drinking water treatment and electric power generation facilities. Environ. Manag..

[14] Fantle-Lepczyk JE (2022). Economic costs of biological invasions in the United States. Sci. Total Environ..

[15] Vander Zanden MJ, Olden JD (2008). A management framework for preventing the secondary spread of aquatic invasive species. Can. J. Fish. Aquat. Sci..

[16] Haight RG, Kinsley AC, Kao S-Y, Yemshanov D, Phelps NBD (2021). Optimizing the location of watercraft inspection stations to slow the spread of aquatic invasive species. Biol. Invasions.

[17] Hammond D, Ferris G (2019). Low doses of Earthtec QZ ionic copper used in effort to eradicate quagga mussels from an entire Pennsylvania lake. Manag. Biol. Invasions.

[18] LimnoTech. Good Harbor Bay dreissenid mussel control demonstration project. Final project report (2020).

[19] Wimbush J, Frischer ME, Zarzynski JW, Nierzwicki-Bauer SA (2009). Eradication of colonizing populations of zebra mussels (*Dreissena polymorpha*) by early detection and SCUBA removal: Lake George, NY. Aquat. Conserv. Mar. Freshw. Ecosyst..

[20] Hargrave, J. & Jensen, D. Assessment of the water quality conditions at Ed Zorinsky Reservoir and the zebra mussel (*Dreissena polymorpha*) population emerged after the drawdown of the reservoir and management implications for the District's Papillion and Salt Creek Reservoirs. https://apps.dtic.mil/sti/pdfs/ADA581189.pdf (2012).

[21] Leuven RSEW, Collas FPL, Koopman KR, Matthews J, Velde GVD (2014). Mass mortality of invasive zebra and quagga mussels by desiccation during severe winter conditions. Aquat. Invasions.

[22] Molloy DP (1998). The potential for using biological control technologies in the management of *Dreissena* spp. J. Shellfish Res..

[23] Kirk, J. P., Killgore, K. J. & Sanders, L. G. Potential of North American molluscivorous fish to control dreissenid mussels. https://erdc-library.erdc.dren.mil/jspui/bitstream/11681/4708/1/ZMR-VOL-1-1.pdf (2001).

[24] Reynolds JD, Donohoe R (2001). Crayfish predation experiments on the introduced zebra mussel, *Dreissena polymorpha*, in Ireland, and their potential for biocontrol. Bull. fr. pêche piscic.

[25] Whitledge G (2015). An evaluation Zequanox® efficacy and application strategies for targeted control of zebra mussels in shallow-water habitats in lakes. Manag. Biol. Invasions.

[26] Luoma J, Severson TJ, Barbour MT, Wise JK (2018). Effects of temperature and exposure duration on four potential rapid-response tools for zebra mussel (*Dreissena polymorpha*) eradication. Manag. Biol. Invasions.

[27] Luoma J, Severson T, Wise J, Barbour M (2018). Exposure-related effects of Zequanox on juvenile lake sturgeon (*Acipenser fulvescens*) and lake trout (*Salvelinus namaycush*). Manag. Biol. Invasions.

[28] Watters A (2011). Effectiveness of EarthTec ® on killing invasive quagga mussels (*Dreissena rostriformis bugenis*) and preventing their colonization in the Western United States. Biofouling..

[29] Watters A, Gerstenberger SL, Wong WH (2013). Effectiveness of EarthTec® for killing invasive quagga mussels (*Dreissena rostriformis bugensis*) and preventing their colonization in the Western United States. Biofoul. J. Bioadhes. Biofilm Res..

[30] Claudi, R., Prescott, T., Mastisky, S. & Coffey, H. *Efficacy of copper based algaecides for control of quagga and zebra mussels*. 58 www.rntconsulting.net (2014).

[31] *Federal Insecticide, Fungicide, and Rodenticide Act (FIFRA): Section 24**(c)*.

[32] *Federal Insecticide, Fungicide, and Rodenticide Act (FIFRA): Section 18*.

[33] Lund K, Cattoor KB, Fieldseth E, Sweet J, McCartney MA (2018). Zebra mussel (*Dreissena polymorpha*) eradication efforts in Christmas Lake, Minnesota. Lake Reservoir Manag..

[34] Murray-Gulde CL, Heatley JE, Schwartzman AL, Rodgers JH (2002). Algicidal effectiveness of Clearigate, Cutrine-Plus, and copper sulfate and margins of safety associated with their use. Arch. Environ. Contam. Toxicol..

[35] Brix KV, Esbaugh AJ, Grosell M (2011). The toxicity and physiological effects of copper on the freshwater pulmonate snail, *Lymnaea stagnalis*. Comp. Biochem. Physiol. C Toxicol. Pharmacol..

[36] Basile A (2012). Toxicity, accumulation, and removal of heavy metals by three aquatic macrophytes. Int. J. Phytorem..

[37] Waller DL (1993). Toxicity of candidate molluscicides to zebra mussels (*Dreissena polymorpha*) and selected nontarget organisms. J. Great Lakes Res..

[38] Fernald, R. T. & Watson, B. T. Eradication of zebra mussels (*Dreissena polymorpha*) from Millbrook Quarry, Virginia: Rapid response in the real world. in *Quagga and Zebra mussels: Biology impacts and control* 195–213 (CRC Press LLC, 2014).

[39] Densmore, C. L. *et al. An evaluation of the toxicity of potassium chloride, active compound in the molluscicide potash, on salmonid fish and their forage base*. 46 https://pubs.er.usgs.gov/publication/ofr20181080 (2018).

[40] Barbour MT, Wise JK, Luoma JA (2018). A bioassay assessment of a zebra mussel (*Dreissena polymorpha*) eradication treatment. Open-File Report.

[41] Luoma, J. A. *et al.* Assessment of uncontained Zequanox applications For zebra mussel control in a Midwestern lake. *Open-File Report.*10.3133/ofr20191126 (2019).

[42] Charles E. S. *et al.* Guidelines for deriving numerical national water quality criteria for the protection of aquatic organisms and their uses. *US Environmental Protection Agency.* https://semspub.epa.gov/work/10/500014779.pdf (1985).

[43] Kamrin, M. A. Pesticide profiles: Toxicity, environmental impact, and fate. (CRC Press LLC, 1997).

[44] Siemering GS, Hayworth JD, Greenfield BK (2008). Assessment of potential aquatic herbicide impacts to California aquatic ecosystems. Arch. Environ. Contam. Toxicol..

[45] Weber, M. M. Zequanox application technique pilot study on Lake Erie. https://invasivemusselcollaborative.net/wp-content/uploads/2018/11/Zequanox-Application-Strategy-Pilot-on-Lake-Erie-Final-Report-FINAL.pdf (2015).

[46] Wagner JL, Townsend AK, Velzis AE, Paul EA (2017). Temperature and toxicity of the copper herbicide (NautiqueTM) to freshwater fish in field and laboratory trials. Cogent Environ. Sci..

[47] Rosenberg G, Ludyanskiy ML (1994). A nomenclatural review of *Dreissena* (Bivalvia: Dreissenidae), with identification of the quagga mussel as *Dreissena bugensis*. Can. J. Fish. Aquat. Sci..

[48] De Ventura L, Kopp K, Seppälä K, Jokela J (2017). Tracing the quagga mussel invasion along the Rhine River system using eDNA markers: Early detection and surveillance of invasive zebra and quagga mussels. MBI.

[49] Sepulveda A (2019). Using environmental DNA to extend the window of early detection for dreissenid mussels. MBI.

[50] Goldberg CS (2016). Critical considerations for the application of environmental DNA methods to detect aquatic species. Methods Ecol. Evol..

[51] Thomsen PF, Willerslev E (2015). Environmental DNA: An emerging tool in conservation for monitoring past and present biodiversity. Biol. Conserv..

[52] Metcalfe-Smith JL, Di Maio J, Staton SK, Mackie GL (2000). Effect of sampling effort on the efficiency of the timed search method for sampling freshwater mussel communities. J. N. Am. Benthol. Soc..

[53] Pooler PS, Smith DR (2005). Optimal sampling design for estimating spatial distribution and abundance of a freshwater mussel population. J. N. Am. Benthol. Soc..

[54] Ferguson JM, McCartney MA, Blinick NS, Schroeder L, Fieberg J (2019). Using distance sampling to estimate densities of zebra mussels (*Dreissena polymorpha*) in early-stage invasions. Freshw. Sci..

[55] Galloway A (2022). Predicting dreissenid mussel abundance in nearshore waters using underwater imagery and deep learning. Limnol. Oceanogr. Methods.

[56] Walters C (1986). Adaptive Management of Renewable Resources.

[57] McLain RJ, Lee RG (1996). Adaptive management: Promises and pitfalls. Environ. Manag..

[58] Santore RC, Di Toro DM, Paquin PR, Allen HE, Meyer JS (2001). Biotic ligand model of the acute toxicity of metals. 2. Application to acute copper toxicity in freshwater fish and Daphnia. Environ. Toxicol. Chem..

[59] Paquin PR, Santore RC, Wu KB, Kavvadas CD, Di Toro DM (2000). The biotic ligand model: A model of the acute toxicity of metals to aquatic life. Environ. Sci. Policy.

[60] Paquin PR (2002). The biotic ligand model: A historical overview. Comp. Biochem. Physiol. C Toxicol. Pharmacol..

[61] *US EPA.* Reregistration eligibility decision (RED) for coppers. https://archive.epa.gov/pesticides/reregistration/web/html/index-71.html (2009).

[62] Mastin BJ, Rodgers JH (2000). Toxicity and bioavailability of copper herbicides (Clearigate, Cutrine-Plus, and copper sulfate) to freshwater animals. Arch. Environ. Contam. Toxicol..

[63] Boutin C, Freemark KE, Keddy CJ (1995). Overview and rationale for developing regulatory guidelines for nontarget plant testing with chemical pesticides. Environ. Toxicol. Chem..

[64] Jones HP (2016). Invasive mammal eradication on islands results in substantial conservation gains. Proc. Natl. Acad. Sci. USA.

[65] Moffitt, C. M., Barenberg, A., Stockton, K. A. & Watten, B. J. Efficacy of Two Approaches for Disinfecting Surfaces and Water Infested with Quagga Mussel Veligers. *Biol. Manag. Invasive Quagga Zebra Mussels West. United States* 467–478 (2015).

[66] Stockton-Fiti, K. & Moffitt, C. Investigation of the Edwards protocol’s effectiveness on dreissenid mussel veligers. https://kasf.fiti.net/s/Report-Edward-protocol-Dreissenid-veligers-Final.pdf (2017).

[67] Marrone Bio Innovations. Zequanox® label. https://marronebio.com/download/zequanox-label/ (2019)

[68] Meehan S, Shannon A, Gruber B, Rackl SM, Lucy FE (2014). Ecotoxicological impact of Zequanox®, a novel biocide, on selected non-target Irish aquatic species. Ecotoxicol. Environ. Saf..

